# Stress in dipteran insects mass‐reared for sterile insect technique applications

**DOI:** 10.1111/1744-7917.70057

**Published:** 2025-06-06

**Authors:** Caroline K. Mirieri, Vera I.D. Ros, Adly M.M. Abd‐Alla, Monique M. van Oers

**Affiliations:** ^1^ Laboratory of Virology Wageningen University and Research Wageningen The Netherlands; ^2^ Insect Pest Control Laboratory, Joint FAO/IAEA Programme of Nuclear Techniques in Food and Agriculture International Atomic Energy Agency, Vienna International Centre Vienna Austria

**Keywords:** Diptera, immunity, immune response, mass‐rearing, SIT, stress response

## Abstract

Stress may be viewed as the disturbance of homeostasis of an organism. Stress may arise from the external or internal environment of living organisms and plays a significant role in the fight or flight responses of animals. An organism's potential to resist stress is determined by its ability to mount up an effective response against the stress factors. Therefore, stress‐induced biomolecules are useful indicators of a well‐functioning immune system. While the interactions between stress and immunity are well‐studied in vertebrate and plant systems, they are insufficiently documented among invertebrates, including dipteran insects that are mass‐reared for sterile insect technique (SIT) applications. Generally, mass‐reared insects may experience a variety of stress factors, which may affect various biological traits, including fecundity, weight of the progeny, adult emergence rates, flight propensity, mating ability, and their competitiveness with wild conspecifics. Many of these biological traits determine the costs and success of SIT programs. It is imperative to understand how stress impacts the quality of the reared insects and their biological traits, as well as the insect's defense responses to stress factors, to maintain robust and healthy colonies for successful release programs in SIT. Here, we review and discuss the sources and responses to biotic and abiotic stress in general in insects, while prioritizing literature on dipteran insects for SIT programs. We also coalesce genes and pathways that are modulated during stress and may be used as indicators to diagnose stress with the final aim to improve insect health in mass‐rearing colonies.

## Graphical abstract

The connections between biotic and abiotic stress affecting mass‐reared dipteran insects and the associated stress and immunological responses. Numbers indicate the order in which the topics are discussed in this review.

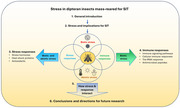

## Introduction

Mass‐rearing of insects is often done for beneficial purposes including: (a) securing human and animal nutrition through food and feed by rearing insects such as crickets (Weissman *et al.*, 2012; Feng *et al.*, 2018), mealworms (Vigneron *et al.*, 2019), and black soldier flies (Feng *et al.*, 2018); (b) contributing to plant health and survival such as in the use of insect frass as fertilizers (Beesigamukama *et al.*, 2020; Houben *et al.*, 2020), the action of insects as pollinators (e.g., honey and bumble bees) (Valido *et al.*, 2019), and the use of insects as natural enemies (predators and parasitoids) against insect pests (Otsuki & Yano, 2019; Wang *et al.*, 2019); (c) providing fabric and clothing (silkworms) (Rahmathulla, 2012; Jiang, 2021); (d) producing antimicrobial peptides (AMPs) for pharmaceutical use, as potentially found in black soldier flies among other insects (Albiol‐Matanic & Castilla, 2004; Yi *et al.*, 2014; Moretta *et al.*, 2020); (e) contributing to environmental sustainability as reported for mealworms that are able to degrade polystyrene and plastic waste (Brandon *et al.*, 2018; Vigneron *et al.*, 2019) and last but not least; (f) managing insect vectors/pests through suppression and eradication of wild insect populations using the sterile insect technique (SIT) (e.g., Vreysen, 2001), on which this review will focus.

Some of the dipteran insects managed by suppression and eradication using SIT are vectors of pathogens threatening human and livestock health through morbidity and mortality (Pant, 1987; Fèvre *et al.*, 2008; Thompson *et al.*, 2020). Other dipterans are pests that cause severe damage in agriculture (Soliman, 2012; Stephenson *et al.*, 2020), resulting in huge economic losses (Swallow, 2000; Enserink, 2007; Muhanguzi *et al.*, 2017; Abro *et al.*, 2021a, 2021b) and thereby also threatening nutrition and food security.

Consequently, there is need for implementing diverse methods of dipteran control to reduce the impact of these vectors/pests, through the application of chemicals, biopesticides, biological control agents (e.g., predators and parasitoids), *Wolbachia*‐induced cytoplasmic incompatibility, and the use of SIT (Vreysen *et al.*, 2000; Zabalou *et al.*, 2004; Alemu *et al.*, 2007; Enserink, 2007; Abro *et al.*, 2021). SIT is normally applied as part of area‐wide integrated pest management (AW‐IPM) approaches, where entire populations of (often dipteran) insects are targeted for suppression, using a combination of control methods such as chemical spraying and trapping, before the final step of eradication, containment or prevention, using sterile male insects (e.g., Vreysen, 2001; Dyck *et al.*, 2005; Vreysen *et al.*, 2013). Sterile males are released in target areas to outcompete wild males in mating with virgin females in the field, resulting in dominant lethal mutations in embryos and hence, nonviable progeny. Sterile males are sequentially released over a relative long period of time and at higher numbers than their wild competitors, and consequently, the target field populations are diminished (Vreysen, 1995; Vreysen, 2001; Dyck *et al.*, 2005; Benedict, 2021). The first and most successful SIT‐based eradication effort was that of the screw worm *Cochliomyia hominivorax* (Coquerel) (Diptera: Calliphoridae) in North America in 1957, followed by Mexico in 1991, Belize in 1992, Guatemala in 1993, and El Salvador in 1994 (Galvin & Wyss, 1996; Wyss, 2000). SIT was successfully employed in eradicating tsetse flies (family Glossinidae) and tephritid fruit flies (Tephritidae) (Abdel‐Malek *et al.*, 1966; Rohwer, 1987; Msangi *et al.*, 1998). Projects are ongoing to develop SIT for control of *Drosophila suzukii* (Matsumara) (Diptera: Drosophilidae) (FAO/IAEA, 2022).

SIT requires the mass‐rearing of flies in large factories, which are then sterilized in the pupae stage or adults using ionizing radiation (gamma or X‐rays) before release as adults in target areas (Chambers, 1977; Schwarz *et al.*, 1985; Benedict *et al.*, 2009; Parker *et al.*, 2021), and it is during all these processes that the insects are exposed to stress factors (Rull *et al.*, 2012), since the organisms live under nonnatural conditions and often at high densities. High densities may lead, to increased food competition, aggressive behavior and, ultimately, cannibalism. As such, the (partially) controlled but unnatural environments create imbalance in the internal status quo of the mass‐reared organisms, thereby favoring stress (Rosche *et al.*, 2021a). To minimize stress for dipteran insects for SIT, standard operating protocols have been developed for mass‐rearing, irradiation, transport, and release, along with quality control tests (Schwarz *et al.*, 1985; Calkins & Parker, 2005; Parker & Mehta, 2007; FAO/IAEA, 2012, 2019, 2020; Culbert *et al.*, 2020).

In this review, sources of stress refer to any factors that negatively impact the insects, whether caused by interactions with their nonliving environment or with other living agents. As such, we speak about biotic and abiotic stress factors, respectively. Abiotic stress may arise from unnatural environmental or treatment circumstances, such as aberrant temperatures, high CO_2_ levels, extreme humidity, or insufficient water supply (Teshome *et al.*, 2020). Dipterans reared for SIT are being exposed to abiotic stressors that include unnatural temperatures, mechanical shocks, and irradiation aimed at sterilization. These abiotic stress factors and their consequences will be discussed in detail in section *Major sources of stress in dipterans for SIT*. Biotic stress arises from living entities such as predators, parasites, or microorganisms (Teshome *et al.*, 2020). In the SIT‐related mass‐rearing of insects, viruses, bacteria, and fungi are the most likely causes of biotic stress. Examples are the viral infections reported in tsetse fly species, such as the Glossina pallidipes salivary gland hypertrophy virus (GpSGHV) and more recently the Glossina morsitans morsitans iflavirus (GmmIV) and Glossina morsitans morsitans negevirus (GmmNegV) (Abd‐Alla *et al.*, 2010; Meki *et al.*, 2021; Mirieri *et al.*, 2023). The first published virus infecting tephritid flies, named cytoplasmic inclusion virus, was found in a *Bactocera tryoni* (Froggatt) (Diptera: Tephritidae) laboratory population (Moussa, 1978). Recently, a large diversity of viruses that included 34 putative RNA viruses of 8 families were found across 9 tephritid fruit fly species, raising potential health concerns for mass‐reared fruit flies (Sharpe *et al.*, 2021). A bit later, 13 RNA viruses were detected in the medfly, *Ceratitis capitata* (Wiedemann) (Diptera: Tephritidae), alone (Hernández‐Pelegrin *et al.*, 2022). Mosquito‐specific viruses have also been discovered, although the presence and possible impact on the health of (mass‐reared mosquitoes is yet to be determined (Guégan *et al.*, 2018). Duguma *et al.* (2017) showed that the addition of protozoan ciliates and rotifers to containers, in which *Culex nigripalpus* (Theobald) (Diptera: Culicidae) mosquitoes were reared, retarded the development of these mosquitoes.

Stress responses are generally associated with abiotic stressors, while the immune response is associated with biotic stressors, although both responses are often linked in their origin and response (Ottaviani & Franceschi, 1997, 1998). According to Selye (1956), in a synoptic view of the stress mechanism, the stress response system in both vertebrates and invertebrates involves the hormonal response system with (neuro‐)endocrine components, as well as the immune system. Acute forms of stress that last for a short period may be directly overcome through cellular and physiological mechanism, or simply by a behavioral response, while chronic forms of stress may lead to a cascade of biochemical and molecular biological events that may over time lead to a lowered resistance to pathogens (Aruoma, 1998; Ho *et al.*, 2013; Frijhoff *et al.*, 2015). On the other hand, mild forms of stress can have beneficial, hormetic effects on an individual including improved resistance to pathogens. An example is the increased resistance of the wax moth *Galleria mellonella* (Linnaeus) (Lepidoptera: Pyralidae) to entomopathogenic fungi following a 30‐min mild heat‐shock exposure (Wojda *et al.*, 2009). Stimulatory effects on the third instar of the predatory, spined soldier bug *Podisus distinctus* (Stål) (Hemiptera: Pentatomidae) to sublethal doses of topically applied pyrethroid permethrin were witnessed as increases in survival time and fertility of the insects (Guedes *et al.*, 2009). Stress can originate from a wide range of environmental conditions encountered by insects, but given that insects belong to the most abundant and diverse class in the animal kingdom, it is clear that they are well able to face and overcome numerous stressors in those environments (Sheehan *et al.*, 2020). This is further elaborated on in section *The stress response* and *The immune response*.

**Fig. 1:**
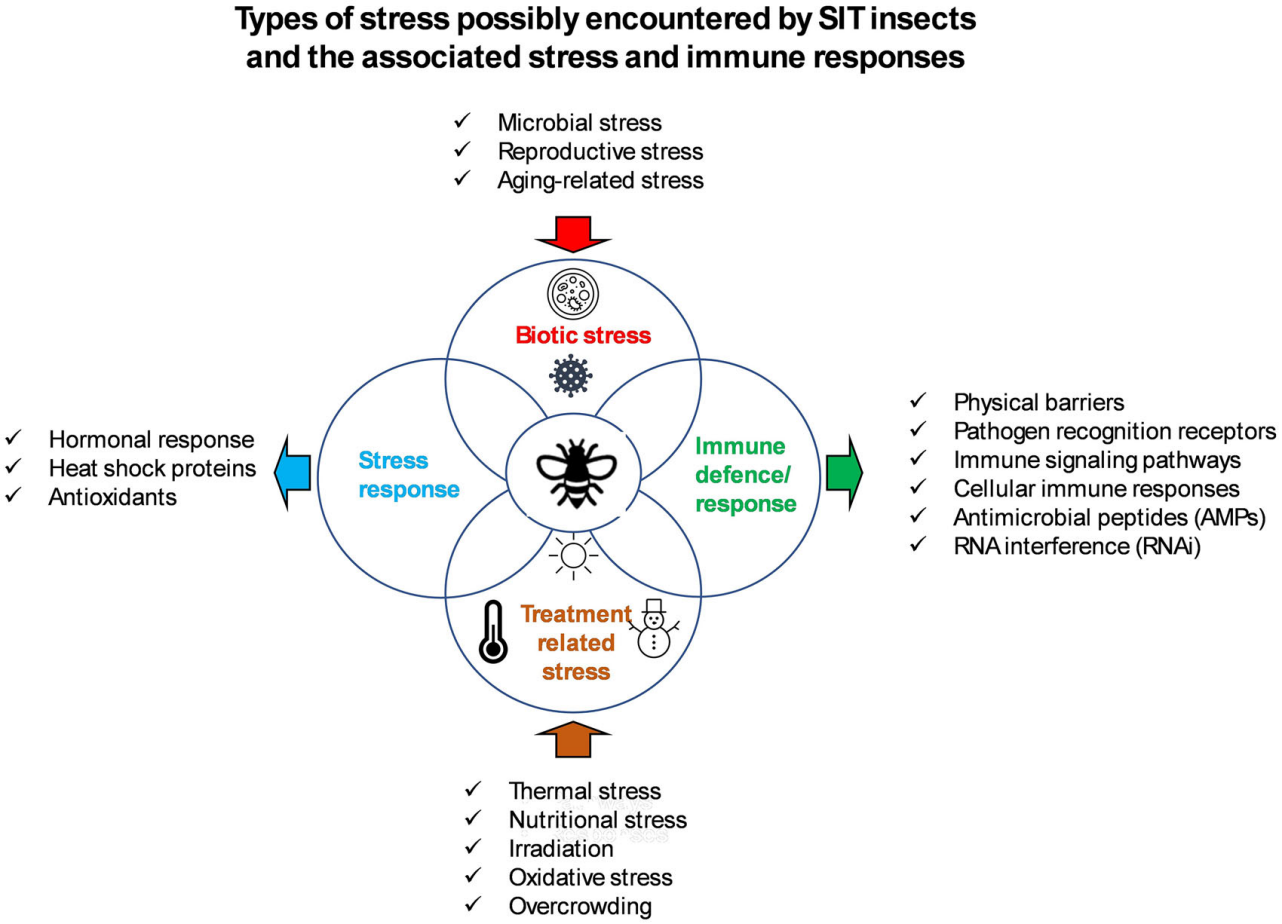
A schematic diagram showing the types of stress and the associated stress and immune responses in insects as discussed in this review, with a focus on mass‐reared dipteran insects for SIT.

Numerous studies have investigated immune responses to pathogenic infections in insects, but the stress response, which is imperative for beneficial and/or colonized insects in their varying environments, received much less attention. Stress and immune response studies in relation to the health of mass‐reared insects for SIT, especially focusing on the underlying molecular mechanisms in these often dipteran species, are also limited. However, meeting the demand of high‐quality insects for cost‐effective SIT programs necessitates an in‐depth understanding of the effects of biotic and abiotic stress factors during mass‐rearing/mass‐production and from there, all the way to the point of insect release into the field (Culbert *et al.*, 2020; Zhang *et al.*, 2020). In this review, we therefore aim to provide an overview of the stress factors acting on dipteran insects used for SIT, coupled with the associated stress/immune responses with examples from tsetse flies, fruit flies, and mosquitoes. Fig. 1 provides a schematic overview of the content of this review. In cases where certain stressors are likely to affect the dipteran insects used for SIT, but relevant literature is not available, examples from other insects are presented and the potential effects are discussed for dipteran insects used in SIT programs.

## Major sources of stress in dipterans for SIT

### Introduction to sources of stress and their effect on insect quality and performance

Stress during the processing stages of mass‐rearing of dipteran insects for SIT can negatively affect their biological quality and performance. Performance may be demonstrated by several parameters, including fertility, number of offspring produced, weight of pupae, emergence rates, flight propensity, mating behavior, insemination rates, and survival (Zuk, 1987; Pagabeleguem *et al.*, 2016; Poda *et al.*, 2018). The effect on biological quality may further be evident when metabolic rates and neuro‐endocrine reactions that modulate immunity are altered (Ardia *et al.*, 2012). Such changes are often linked to changes in the expression of genes, which in turn may serve as markers for the type and level of stress (Harrington, 2011). An overview of response pathways and genes that may be used as markers of stress and which could be explored for prophylactic/therapeutic targets to manage stress in dipteran insects reared for SIT purposes is provided on Table 1. Determining which circumstances are causing stress and how these affect the health of these captured and mass‐rearing‐adapted insects is necessary to avoid suboptimal conditions in order to ensure success of the sterile insects in the field and to prevent the collapse of reared colonies, which lead to interruption of their intended use and cause large economical set‐backs for the rearing facilities (Alemu *et al.*, 2007; Abd‐Alla *et al.*, 2012).

**Table 1:** A collation of induced pathways and activated genes in insects by various stress factors and microbes as sourced from various literature

	Response/pathways	Associated genes, proteins	Causative factor(s)	Insect examples	Selected references
Stress responses	Phenoloxidase pathway, antioxidant defense pathway	PO cascade enzymes: prophenoloxidase (*ppo‐2*), its activating enzyme (*ppae‐1*), free‐radical scavenging enzymes‐ superoxide dismutase (*sod*) and catalase (*cat*)	Irradiation	*Spodoptera litura*	Sachdev *et al.*, 2017
	Oxidative stress response, Antioxidant pathways	Oxidative stress markers, Fe/Mn sod, Cu/Zn sod,, sod3 cat 1 and 2, peroxiredoxin, thioredoxin peroxidase 1, thioredoxin reductase, glutathione peroxidase, antioxidant enzymes in general	Oxidative stress, reproductive stress (e.g., parturation, lactation and involution)	*Glossina morsitans morsitans*	Michalkova *et al.*, 2014
	Prostaglandin, eicosanoid biosynthesis pathway	Heat shock proteins, GST‐2, Bax inhibitor and P450(high temp)‐PGRP‐LB, antimicrobial peptides (AMPs), lysozyme, serine protease, small Hsp's and Hsp90, ferritin, superoxide dismutase (SOD), GSHt, oxidized glutathione (GSSG)	Temperature stress: heat shock, chilling, cold stress	*Drosophila melanogaster*, coleopteran insects, various insects	Morrow *et al.*, 2004; Lalouette *et al.*, 2011; Wojda, 2017; Chen *et al.*, 2019
	The unfolded protein response (UPR)	PERK (PKR‐like ER kinase), ATF6 (activating transcription factor 6), IRE1*α* (inositol‐requiring enzyme 1*α*)	Oxidative stress, changes in temperature or pH, lack of nutrients, viral infections	Arthropods in general, *D. melanogaster*	Rosche *et al.*, 2021b
	The integrated stress response (ISR)	*GCN2*, (PKR‐like ER kinase), HRI (heme‐regulated inhibitor), PKR (protein kinase double‐stranded RNA‐dependent)	UV irradiation, nutrient deprivation, accumulation of misfolded proteins in the ER‐(causing ER stress), oxidative stress, iron deficiency, viral and bacterial infections	Arthropods in general, *D. melanogaster*	Rosche *et al.*, 2021b
	The Jun N‐terminal kinase (JNK) signaling pathway, antioxidants	CG5002, dGCC185 and GstS1, mitochondrial Hsp22, mitochondrial proteins, genes of the OXPHOS system, *hsp26* or *hsp27*, *mth* gene	Longevity	*D. melanogaster*, *Spodoptera frugiperda*, *Spodoptera exigua*	Morrow *et al.*, 2004; Wang *et al.*, 2004; Kim & Kim, 2010
	Antimicrobial peptides (AMPs) produced by hemocytes	Attacins, cecropins, defensins	Nutritional stress	*Glossina* species, *D. melanogaster*, *Eupoecilia ambiguella*	Akoda *et al.*, 2009; Vogelweith *et al.*, 2015b; Rosche *et al.*, 2021b
Immune responses	AMPs	Defensins, thanatin, gloverins, proline‐rich peptides, lysozyme	Gram positive bacteria, plasmodium, fungi, yeast	*Hermetia illuscens*, Insects in general	Bahar & Ren, 2013; Bruno *et al.*, 2021
		Drosomycin (via Toll), thanatin, diptecirin (via Imd)	Bacteria, fungi	*D. melanogaster*, various insects	Bahar & Ren, 2013; Kingsolver *et al.*, 2013
		Defensins, cecropins, attacins, lebocins, proliner‐rich peptides, gloverins, moricins, plasma or hemocyte dopachrome conversion enzyme, drosocin, diptericin	Bacteria, fungi, viruses, parasites	lepidopteran insects, *H. illuscens*	Yi *et al.*, 2014; Wu *et al.*, 2018
	Complement related proteins	Macroglobulin complement‐related factor (AaMCR), Savenger receptor‐C (AaSR‐C)	Virus infection	*Aedes aegypti*	Xiao *et al.*, 2014
	RNA interference (RNAi)	Argonaut, Dicer, Drosha (*ago1*, *ago2*, *dcr2*, micro RNAs, siRNAs, piRNA, Piwi‐proteins	Viral infections	*Ae. aegypti*, *Glossina pallidipes*	Lucas *et al.*, 2013; Meki *et al.*, 2018
		siRNA, Dicer‐2, R2D2, Ago2	Viral infections	*D. melanogaster*	Kingsolver *et al.*, 2013
		piRNA: Piwi, Aubergine, Ago3	Transposons, viral infections	*D. melanogaster*, Culicidae (*Aedes* spp.)	Miesen *et al.*, 2015, 2016; Gamez *et al.*, 2020
		MicroRNAs, Dicer‐1, Ago1, Centrosomal protein (*CEP*), Fibrillin‐1 (*FBN1*), Ras‐related protein (*Ral‐a*), *Hipk*, *Rab27*, *Apoltp*, Transcription factor collier (*TFCOE*), serine, threonine kinase protein (*STKP*), Twitchin, Vitellogenin receptor (*VtgR*)	Infections, developmental changes	*G. pallidipes*	Meki *et al.*, 2018
	Melanization	Dopachrome conversion enzyme (DCE), Dopa decarboxylase (DDC), phenylalanine hydroxylase (PAH), phenoloxidase (PO), and tyrosine hydroxylase (TH)	Pathogen infection (viruses, bacteria, parasites)	*D. melanogaster*, Culicidae, *Galleria mellonella*	Christensen *et al.*, 2005; Nappi *et al.*, 2009; Sheehan *et al.*, 2020
	Cellular responses	Phagocytoses, capsule formation, nodule formation, RHG genes (*reaper*, *hid*, *grim*, and *sickle*)	Pathogens, viral infections	*D. melanogaster*, various dipterans, various lepidopterans	Goyal *et al.*, 2000; Lavine & Strand, 2002; Lamiable & Imler, 2014; Rosales, 2017
	Imd pathway	*relish* (*Rel 2*), dorsal and dorsal‐related immune factor (*dif*), antiviral peptides	Viral infections	Dipteran insects, *Bombyx mori*	Lin *et al.*, 1998; King & Macrae, 2015
		Peptidoglycan recognition protein‐LC (PGRP‐LC) and PGRP‐LE, IMD (adaptor protein), FADD, DREDD, *relish*, PGRP‐SB1 and PGRP‐SD	Bacteria	*D. melanogaster*, *Aedes* spp., *Culex* spp., various other insects	Rosales, 2017; Palmer *et al.*, 2018
	Toll pathway	TLRs, cytoplasmic Toll, IL‐1R (TIR), leucine‐rich repeats (LRRs), AMPs (Drosomycin) *dorsal*, *dif*, *spatzle*, *myD88*, *rel1*	Bacteria, fungi	*D. melanogaster*, *G. morsitans morsitans*	Lin *et al.*, 1998; MacLeod *et al.*, 2007b
		PGRP‐SA, PGRP‐SD, GNBP‐1, GNBP‐3, *spätzle*, toll receptors, *dMyD88*, *tube and pelle*, *cactus*, *dif*, *dorsal*	Gram‐positive bacteria and fungi	*D. melanogaster*, Culicidae (*Aedes* spp., *Anopheles* spp., *Culex* spp.), *S. frugiperda*	Kingsolver *et al.*, 2013; Cheng *et al.*, 2016
	JAK‐ STAT pathway	*vago*, *vir‐1*, *hopscotch*, *domeless* and 3‐unpaired‐related ligands (*upd 3*), phosphorylated STATs	Pathogen infection – viruses (e.g., DENV and ZIKV), bacteria, nutritional stress	*D. melanogaster*, Culicidae (*Aedes* spp., *Anopheles* spp., *Culex* spp.), *S. frugiperda*	Kingsolver *et al.*, 2013; Lamiable & Imler, 2014; Cheng *et al.*, 2016
	Autophagy pathway	Phosphoinositide 3‐kinase (P13K), Akt pathway, TOR, Atg13, Atg1, Atg13 phosphatase	Pathogen infection – viruses (DENV and ZIKV), bacteria, fungi, parasites, nutritional stress	*D. melanogaster*	Rosales, 2017

**Fig. 2:**
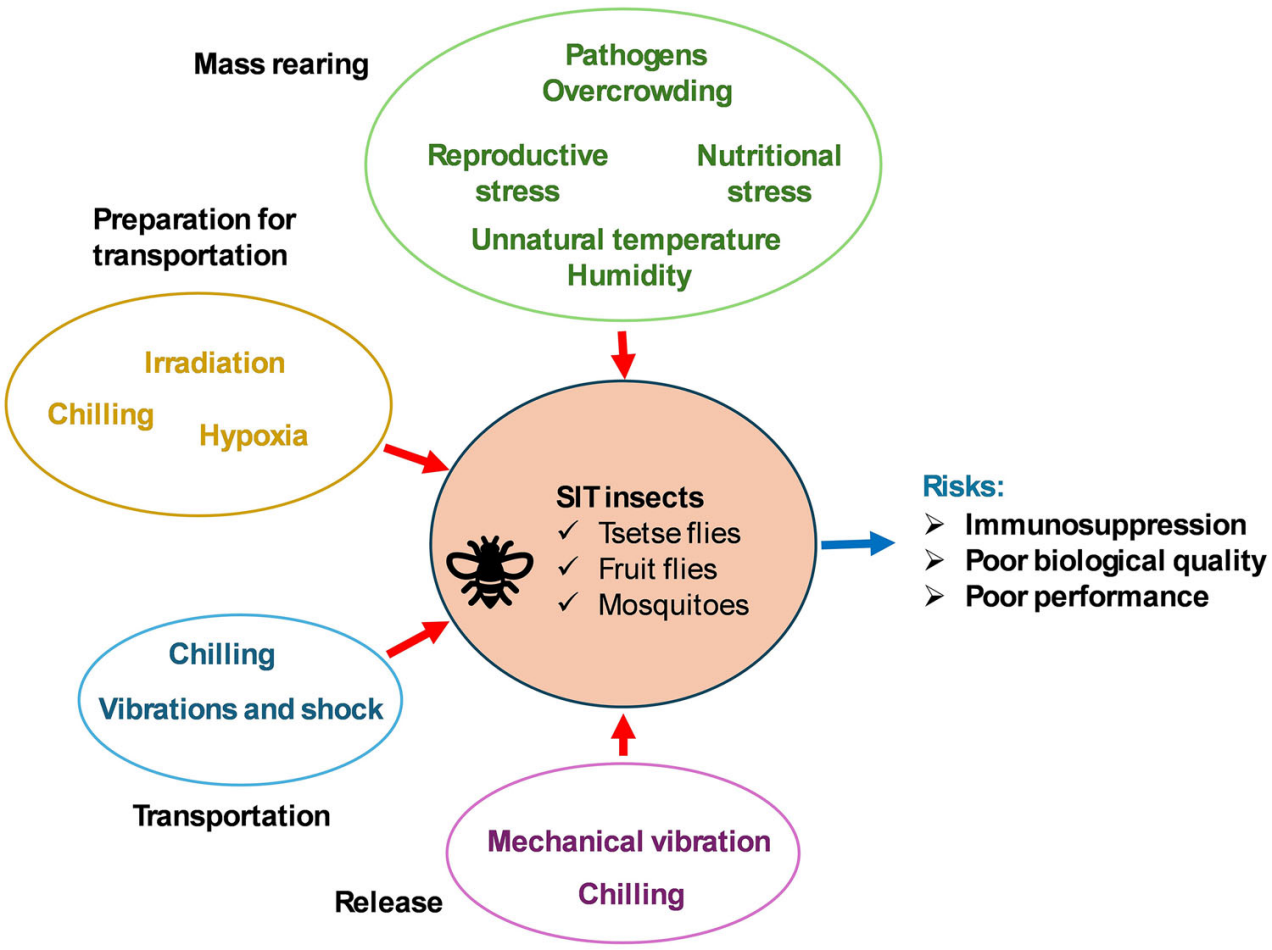
An illustration of the sources of stress acting during different phases of dipteran insect production and release for SIT. The diagram shows how these factors may affect SIT insects (tsetse flies, fruit flies and mosquitoes), resulting in immunosuppression, poor biological quality and suboptimal performance.

During mass‐rearing, likely sources of stress in dipterans for SIT applications originate from production and preparation for release. Depending on the species, this may include nonoptimal temperatures and high humidity in the mass‐rearing facilities, chilling or heating during sex sorting, irradiation and transportation, and side effects of the hypoxia applied to avoid irradiation damage. Furthermore, mechanical impact may be brought about by transportation and the insect release machinery. In addition, dipterans may be infected by pathogenic microbes while in production (Abd‐Alla *et al.*, 2010; Hesketh & Hails, 2010; Mutika & Parker, 2014; Arredondo *et al.*, 2018; Diallo *et al.*, 2019; Mirieri *et al.*, 2020) (Fig. 2). Other sources of stress encountered by insects mas‐reared for SIT may include poor nutrition, cannibalism, too high CO_2_ levels, and high densities while undergoing production (Akoda *et al.*, 2009; Lalouette *et al.*, 2011; Krautz *et al.*, 2014; Sachdev *et al.*, 2017; Wojda, 2017), possibly reducing performance and resilience to pathogens.

### Thermal stress

Temperature is an important factor during mass‐production, irradiation and delivery of sterile insects to their release sites during SIT programs (Mutika *et al.*, 2014, 2019; Diallo *et al.*, 2019; Zhang *et al.*, 2020; Enriquez *et al.*, 2021). It is therefore important to understand the immediate and potential future effects of temperature on the biology of the reared insects. Suboptimal temperature greatly affects biological processes, especially in poikilothermic organisms such as insects, whose internal temperature fluctuates with the external temperature. The average temperature of their natural habitat must be mimicked as much as possible in rearing facilities of insects, for proper functioning and survival of the insects, as large temperature variations can be detrimental (Mutika *et al.*, 2019; Joosten *et al.*, 2020). This regime, however, is most likely also not optimal, as the natural day‐night fluctuations are absent in mass‐rearing facilities.

The effects of the suboptimal temperatures may be acute but there is also evidence that the thermal history of the flies affects their flight behaviour in the filed after release. The medfly *C. capitata* withstood lower field temperatures better when also reared at low temperatures (Esterhuizen *et al.*, 2014). An extensive study exposed pupae of the oriental fruitfly *Bactrocera dorsalis* (Hendel) (Diptera: Tephritidae) for a short time to very high or very low temperatures, and these unnatural temperatures negatively affected many traits, such as reducing fecundity and longevity, and prolonging the preoviposition period (Yu *et al.*, 2022). Recent data indicated that there is a relation between heath stress at young age and autophagy‐based heat‐tolerance of adult *Drosophila melanogaster* (Meigen) (Diptera: Drosophilidae). The underlying mechanism would be that heat‐stress leads to protein aggregation, which in turns triggers a long‐lasting autophagy‐related homoeostatic response (Willot *et al.*, 2023).

Temperature also affects the levels and transmission of pathogens present in insects (Vogels *et al.*, 2016). While there are hardly any studies interrogating the role of temperature stress on the health of mass‐reared dipterans, we can derive examples of potential effects of temperature on such insects from other studies that have vastly demonstrated the effect of temperature on the vector competence of mosquitoes to transmit viruses (Adelman *et al.*, 2013; Alto & Bettinardi, 2013; Bellone & Failloux, 2020; Wimalasiri‐Yapa *et al.*, 2021). Bellone & Failloux (2020) showed that increasing temperatures can lead to increased viral levels in mosquitoes of various species. Natural insect‐specific viral infections have been detected in dipteran species under mass‐rearing, including tsetse flies, tephritid fruit flies, and mosquitoes (Moussa, 1978; Abd‐Alla *et al.*, 2010; Guégan *et al.*, 2018; Meki *et al.*, 2021; Mirieri *et al.*, 2023) under mass‐rearing and may similarly be affected by the various temperatures applied at different stages in preparation for release in SIT programs (Adelman *et al.*, 2013; Alto & Bettinardi, 2013; Bellone & Failloux, 2020; Wimalasiri‐Yapa *et al.*, 2021).

Below, a few examples are given on how variations in temperature can affect the outcome of microbial interactions. In CHIKV‐infected *Aedes aegypti* (Linnaeus) (Diptera: Culicidae), it was found that higher ambient temperatures (28 and 32 °C, compared to 18 °C) correlated with higher virus levels in the mosquitoes (Wimalasiri‐Yapa *et al.*, 2021). Furthermore, temperature was shown to affect parameters important for vectorial capacity (e.g., mosquito densities, biting rates and survival) leading to either increasing or decreasing arbovirus transmission. This was demonstrated in a study, which found that the lifespan of *Ae. aegypti* infected with dengue virus was shorter at 30 °C than at 26 °C (Christofferson & Mores, 2016). Cooler temperatures also increased the survival after arbovirus infection of adults of the Asian tiger mosquito, *Aedes albopictus* (Skuse) (Diptera: Culicidae) and resulted in the lowest rate of arboviral dissemination from these mosquitoes (Alto & Bettinardi, 2013). Microclimates such as those present in natural mosquito breeding sites have also been reported to influence the dynamics of mosquito‐borne viral diseases by affecting the RNA interference (RNAi) antiviral immunity (Adelman *et al.*, 2013). In the same study, cooler circumstances destabilized the RNAi defense system and increased susceptibility of *Aedes* spp. to viral infections. It is therefore evident that temperature affects viruses that infect mosquitoes as well as the mosquitoes themselves. However, the full impact of the rearing temperature on the quality and performance of mass‐reared dipterans for SIT remains largely undetermined.

Effects of temperature in dipterans under mass‐rearing may also be correlated to other stress factors within the same environment. A study showed that, the analyzed immune genes (*βGRP1*, *pelle*, and *relish*) and a number of heat shock protein genes (*Hsps)* were differentially regulated when starved flies were exposed to heat or cold stress, suggesting synergistic effects of nutritional and thermal stress (Blasco‐Lavilla *et al.*, 2021). Also, an investigation on the influence of temperature and humidity combined with nutrient deprivation on the mating and survival of fertile and sterile *D. suzukii* showed that a humidity below 60% impaired mating at low and high temperatures (Krüger *et al.*, 2021). In general the survival was negatively influenced by high temperatures, low humidity, and food deprivation. A study identifying the optimal temperatures and compaction rates for storage and transport of *Ae. aegypti* showed that the mosquitoes survived a wide range of temperatures. The optimal survival level was seen at relatively high compaction rate at lower temperatures (Chung *et al.*, 2018). A temperature rise during long‐distance transportation of *Apis mellifera* (Linnaeus) (Hymenoptera: Apidae) elicited gene and transcriptional responses in the honeybees showing increased methylation and decreased ribosomal and protein folding activity. However, on recovery from transportation, levels of transcripts associated with defense responses, immune activity, and heat shock declined again, implying an impact of high temperatures on stress and immune responses during transport (Melicher *et al.*, 2019).

In CHIKV‐infected *Ae. aegypti* mosquitoes, exposed to 18, 28, or 32 °C, the highest number of differentially expressed host genes were observed at 28 °C, with an upregulation of genes associated with the Toll, IMD (immune‐deficiency), and Janus kinase and “Signal transducers and activators of transcription” (JAK‐STAT pathways), showing that temperature significantly modulated immune gene expression in response to infection (Wimalasiri‐Yapa *et al.*, 2021). In a study evaluating the gene expression of different immune and heat shock genes as influenced by cold and heat in the bumblebee *Bombus terrestris* (Linnaeus) (Hymenoptera: Apidae), Blasco‐Lavilla *et al.* (2021) found that the *β*‐glucan recognition protein 1 (*β*GRP1) receptor gene was upregulated by cold conditions, suggesting increased cellular immunity against bacteria and/or fungi. Insects living in geographical environments with fluctuating seasonal temperatures may exhibit seasonal plasticity in immunity, as exemplified by the changing cuticle color and increase in phenoloxidase levels in crickets reared in simulated summer and fall environments (Fedorka *et al.*, 2013). However, some organisms may be more resilient than others in withstanding thermal stress, as in the example of tsetse flies, which did not show a higher prevalence of GpSGHV infection after exposure to high temperature (Abd‐Alla *et al.*, 2010). Furthermore, mild thermal stress may elicit a positive effect by making insects more resilient through stimulating immune gene expression (Blasco‐Lavilla *et al.*, 2021).

### Irradiation stress

Irradiation is one of the main sources of stress (oxidative stress) in sterile insects and this stress can limit the expected impact of SIT programs due to reduced biological quality of the released insects (Lopez‐Martinez & Hahn, 2012; Lopez‐Martinez *et al.*, 2014). Multiple stressors are often included along with irradiation in SIT programs, exacerbating the impact of irradiation in tsetse flies, fruit flies, and mosquitoes (Curtis & Langley, 1972; Hallinan & Rai, 1973; Diallo *et al.*, 2019; Mirieri *et al.*, 2020; Enriquez *et al.*, 2021; Giustina *et al.*, 2021). In *Ae. albopictus*, gamma irradiation affected the densities of particular bacterial species in the gut microbiome, which may have consequences for the immunity, fitness, and performance of the mosquitoes (Zhang *et al.*, 2021). In the cotton leafworm *Spodoptera litura* (Fabricius) (Lepidoptera: Noctuidae), gamma irradiation upregulated genes important for the phenoloxidase and antioxidant defense mechanisms, implying that this irradiation also challenges the immune system in this insect (Sachdev *et al.*, 2017). In addition, X‐ray treatment was reported to increase the innate resistance of *Ae. aegypti* to infection with the malaria parasite, *Plasmodium gallinaceum*, with the effect increasing quantitatively with higher irradiation doses (Terzian, 1953). Irradiation under hypoxic/anoxic stress also influenced the performance of fruit flies used in SIT programs, where the combination of prolonged hypoxia and gamma irradiation was detrimental (Arredondo *et al.*, 2018; Benelli *et al.*, 2021), while short‐term anoxia during gamma irradiation improved the performance of male flies and moths (Lopez‐Martinez & Hahn, 2012; Lopez‐Martinez *et al.*, 2014). Also, under low oxygen stress (hypoxic conditions) and cold stress, the impact of gamma irradiation was reduced in *D. suzukii* flies (Enriquez *et al.*, 2021). However, gamma irradiation did not affect the virus densities of GmmIV and GmmNegV in *Glossina morsitans morsitans* (Westwood) (Diptera: Glossinidae), as shown in our recent study (Mirieri *et al.*, 2023), where viral densities were similar in irradiated and nontreated control flies.

### Nutritional stress

Nutrition is of great importance to the fitness and performance of insects. Notably, during nutritional deprivation, resources needed for fecundity are often directed toward longevity and survival of insects (Le Bourg & Minois, 2005; Chen *et al.*, 2013). The impact of nutritional quality of larval diet in relation to temperature was compared in *Ae. Aegypti* mosquitoes. For high quality food, a shortened pupal development time was observed with increasing temperatures that led to high teneral energy reserves, consequently leading to increased longevity and tolerance to heat stress of the emerging adults (Sasmita *et al.*, 2019). The lack of food or the poor quality thereof can have a huge impact on the insect's immune system (Vogelweith *et al.*, 2015). During nutrient deprivation, the immune system in general becomes less active (Boersma & Vijverberg, 1996; Siva‐Jothy & Thompson, 2002). For example, the susceptibility of teneral tsetse flies to *Trypanosoma* spp. infestation increased when they had starved compared to fed parents (Akoda *et al.*, 2009). When *D. melanogaster* was parasitized by the parasitoid *Leptopilina boulardi* (Förster) (Hymenoptera, Figitidae) under yeast deprivation, a reduced level of parasitoid egg encapsidation was observed. Provision of yeast‐containing diet 1 d after exposure to the parasitoids had no stimulating effect on the immune response of the flies, demonstrating the importance of this dietary component for the initial immune responsiveness against the parasitoid (Vass & Nappi, 1998). Nutritional deprivation also reduced the ability of mosquitoes to melanize parasites in their midgut (Suwanchaichinda & Paskewitz, 1998). Further, short‐term nutrient deprivation led to a decrease in phenoloxidase activity in the hemolymph of the mealworm beetle *Tenebrio molitor* (Linnaeus) (Coleoptera: Tenebrionidae), but this activity increased soon after food was offered, again implying the important role of nutrition in immunological processes (Siva‐Jothy & Thompson, 2002). Trade‐offs of (nutritional) stress are further discussed in section *Interactions between stress and responses*.

### Oxidative stress, reproductive stress, and age‐related stress

Oxidative stress is linked to exogenous and endogenous stress factors (such as irradiation, hypoxia, reproduction, and infections) that lead to the overproduction of reactive oxygen species (ROS). Oxidative stress occurs when the production of ROS cannot be kept in balance by counteracting antioxidants. The associated changes in the redox status of the cell leads to the production of peroxidases and free radicals. If ROS is not sufficiently detoxified by antioxidants, the redox state of the cell is at stake, and cellular components are damaged through the action of free radicals and peroxidase (Michalkova *et al.*, 2014; Sachdev *et al.*, 2017; Benelli *et al.*, 2021). Oxidative stress is evidently a significant factor during the senescence of social insects: termites, bees, and ants (Kramer *et al.*, 2021). Typically for aging is the diminishing resistance against sources of stress that lead to ROS production, which in the absence of sufficient antioxidants may cause irreparable damage to macromolecules, as shown in *Drosophila* (Le Bourg, 2001). One of the major effects of senescence is low fecundity, and hence, aging is generally avoided in insects reared for SIT programs. Apart from a lower fecundity, performance of SIT insects in the field also declines with age (Abila *et al.*, 2003). Therefore, senescence‐related oxidative stress may be less of an issue here. However, the viviparous tsetse fly *G. morsitans morsitans* was shown to suffer from oxidative stress during the reproductive process (including birth, lactation, and involution) (Denlinger & Ma, 1974; Michalkova *et al.*, 2014). Also, due to oxidative stress caused by depletion of the antioxidant catalase, after blood feeding, the number of developing oocytes in the sandfly *Lutzomyia longipalpis* (Lutz & Neiva) (Diptera: Pyschodidae), decreased (Diaz‐Albiter *et al.*, 2011). Furthermore, infections caused by bacteria and viruses have been shown to contribute to oxidative stress in insects (Wang *et al.*, 2001b; Dubovskiy *et al.*, 2008; Wong *et al.*, 2015).

Conversely, while the production of ROS is often destructive, certain levels of ROS/oxidative molecules may have a positive impact on the immune system of insects. For example, under oxygen deprivation, initial overproduction of ROS led to hypoxic biochemical responses that enhanced antioxidant defenses (Hermes‐Lima *et al.*, 2015; Oliveira *et al.*, 2018). Oxidative stress can also mediate antiviral protection as exhibited in a study where protective *Wolbachia* strains elevated the H_2_O_2_ concentrations and potentially induced oxidative stress in *Drosophila* flies, resulting in reduced susceptibility to Drosophila C virus‐induced (DCV) mortality. In the same study, mutant *Drosophila* flies with higher H_2_O_2_ levels under normal circumstances were found to be less susceptible to DCV mortality, also in absence of *Wolbachia* (Wong *et al.*, 2015). Further, *Wolbachia* can affect the oxidative environment by regulating the ROS levels and hence enhance their survival by preventing their elimination from the host environment (Zug & Hammerstein, 2015).

### Overcrowding stress

Overcrowding has been implicated in triggering and enhancing infection rates of viruses, bacteria, and protozoa (Steinhaus, 1958). Colony size affected thermoregulation and induced temperature stress in bees (Melicher *et al.*, 2019). These infections may arise due to the activation of latent viruses or invasion of micro‐organisms from the surrounding environment into the alimentary tract or due to the close proximity under captivity that allows easy transmission of infectious diseases (Steinhaus, 1958).

In mass‐rearing for SIT, it is essential that crowding in cages and rearing rooms is controlled, to avoid competition for food and space and to reduce the chances of infections and potential collapses of colonies as witnessed in some tsetse fly factories (Abd‐Alla *et al.*, 2011; Kariithi, 2013). Mass‐rearing procedures for SIT dictate the densities of dipterans (of each species of fruit flies, mosquitoes and tsetse flies) within cages, rooms or volumes of water, following several optimization studies, for purposes of crowd control and optimum productivity (Chambers, 1977; FAO/IAEA, 2012, 2019; Balestrino *et al.*, 2014; Kauffman *et al.*, 2017; Desa *et al.*, 2018; 2020; Elaini *et al.*, 2020; Mastrangelo *et al.*, 2021; Pudar *et al.*, 2021). These controlled densities minimize chances of mortality as the insects can move around freely and have sufficient space to feed, for example, for tsetse flies that blood feed on a membrane for a determined duration of time (Pagabeleguem *et al.*, 2021).

On the contrary to the disadvantages of crowding, there may be an advantage to it as density‐dependent prophylaxis has been reported in the mealworm beetle *T. molitor*, where reduced mortality and a higher degree of cuticular melanization were observed when the mealworms were exposed at high densities to a generalist entomopathogenic fungus (Barnes & Siva‐Jothy, 2000). Also, tsetse flies under mass‐rearing for SIT that were colonized in unusually high fly densities (120 to 180 flies/cage) were more susceptible to SGHV infections than low‐density controls (Abd‐Alla *et al.*, 2010; Yimer *et al.*, 2015).

## The stress response

### Introduction to stress responses

Both biotic and abiotic stress can trigger a complex network of standard body responses in organisms. Therefore, stress and immune responses can, in practice, not easily be separated and co‐act in overcoming stressful circumstances. In addition, stress is a crucial driver for a weakened immunity and the corresponding increased susceptibility to pathogenic infections in vertebrates and invertebrates (Ottaviani & Franceschi, 1996). Despite this interconnection, we will discuss in this section typical stress responses to (a)biotic stressors in insects, including the pathways that lead to the production of hormones, heat shock proteins, and antioxidants. In Section *The immune response*, we will discuss response mechanisms and pathways especially directed at counteracting invading microbes. In Section *Interactions between stress and responses*, we come back to the interactions of various stress and immune pathways. Fig. 2 provides an overview of the possible responses to biotic and treatment‐related (mainly abiotic) stress. As already indicated before, Table 1 summarizes pathways and genes/proteins involved in the response to particular (a‐)biotic stressors.

As previously discussed, and as outlined in Table 1, stress factors often occur simultaneously, which may have the following effects on the response of the insect to the stressors: (a) abiotic stressors influencing the occurrence and outcome of biotic stressors or vice‐versa; (b) stressors acting in synergy, either as multiple biotic stressors or (in combination with) abiotic stressors; (c) a single stressor eliciting multiple responses; or (d) multiple stressors eliciting a similar/single response (Adamo, 2012, 2010). Such interactions result in outcomes that have implications on the management of insects during mass‐rearing and post production, for SIT (Kubi *et al.*, 2006; Alto & Bettinardi, 2013; Arredondo *et al.*, 2016, 2018; Sachdev *et al.*, 2017). An example is mechanical stress (including vibrations) acting on mass‐reared SIT flies, which is especially evident in long‐distance transportation of pupae, and has been reported to involve other stress factors, including hypoxia, thermal stress, and suboptimal humidity, which reduce the fitness and performance of, for example, tephritid fruit flies, mosquitoes, and tsetse flies (Chung *et al.*, 2018; Melicher *et al.*, 2019; Benelli *et al.*, 2021; Dominiak & Fanson, 2021).

### Hormonal stress responses

Octopamine (OA) is a neurohormone that is a relative of the biogenic amine norepinephrine (which is generated from tyramine and derived from the amino acid tyrosine) and is released in insects as part of their stress response. Octopamine acts as a signaling molecule during the flight or fight response in insects, resulting in immunosuppression in the event of acute stress (Adamo, 2008). The upregulation of cell immune function in insects and the downregulation of hemocyte numbers due to OA has been demonstrated (Baines & Downer, 1992; Adamo, 2008; Kim & Kim, 2010). Also, OA has immune‐enhancing properties that arise from its ability to maintain optimum function under different physiological conditions (Adamo, 2008). Other hormones in insects that mediate immune functions include 5‐hydroxytryptamine (5‐HT, also named serotonin) and dopamine (DA). The effect of the above three hormones on adenylate cyclase activity was studied using fragmented hemocyte membranes of the cockroach *Periplaneta americana* (Linnaeus) (Blattodea: Blattidae). There was an increased cyclic adenosine monophosphate (cAMP) production from 5‐HT and OA signaling, but DA did not affect adenylate cyclase activity. A 5‐HT increase in cAMP production may result in an alteration of phagocytotic activities of the immune system (Baines & Downer, 1992). Octopamine and 5‐HT also mediated a rapid increase of circulating hemocyte populations of the beet army worm, *Spodoptera exigua* (Hübner) (Lepidoptera: Noctuidae), in response to a bacterial challenge (Kim & Kim, 2010).

In addition, biogenic amines may have crucial roles during the reproduction of insects and associated stress responses. It is supposed that DA and OA act as intermediates between the juvenile hormone (JH) and 20‐hydroxyecdysone (20E) in *Drosophila* reproduction, as investigated in *Drosophila virilis* (Sturtevant) (Diptera; Drosophilidae). An increase in DA led to an increase in 20E and JH, while the absence of OA led to a decrease in 20E and JH levels (Rauschenbach *et al.*, 2007). In tsetse flies, the ecdysteroid hormone peaked preceding parturition (giving birth), suggesting its role in reproduction related stress (Benoit *et al.*, 2018). A study examining the regulation of lipid homeostasis during the transition between lactating and nonlactating periods of tsetse fly pregnancy cycle revealed that the application of the JH analogue methoprene as well as insulin injection in lactating females increased stored lipids and suppressed lipolysis. As a result, the expression of lactation‐specific genes was reduced, leading to elevated larval abortion rates in the pregnant tsetse flies (Baumann *et al.*, 2013). Also, regulation of reproductive processes in mosquitoes has determined 20E as the major hormone regulating egg maturation in females, a process which, if incomplete, can lead to reproductive stress (Roy *et al.*, 2016). Further, β‐ecdyson induced vitellogenesis (accumulation of the yolk in a developing oocyte) in *Ae. aegypti* that were without a blood meal, suggesting that ecdysone can replace the vitellogenin stimulating hormone (VHS) in vitellogenesis, obviously triggered by nutritional stress (Fallon *et al.*, 1974). In *Drosophila*, DA and JH have also been implied as being involved in regulating carbohydrate metabolism (Karpova *et al.*, 2019), as a response to neuroendocrine stress.

### Heat shock proteins stress responses

Heat shock proteins (HSPs), also known as stress proteins, exist in small and large adenosine triphosphate (ATP)‐dependent forms, and function as molecular chaperones in protein folding and transport. They provide stress resistance through prevention of protein denaturation/degradation (King & Macrae, 2015). The functions of individual HSPs differ with developmental stage, subcellular location, and environmental conditions (Jakob *et al.*, 1993; Parsell & Lindquist, 1993; King & Macrae, 2015). The accumulation of misfolded or denatured proteins in the endoplasmic reticulum (ER) leads to ER stress. The unfolded protein response (UPR) may then restore cell homeostasis. In mosquito cells, for example, the UPR was reported to increase the cells’ tolerance to dengue virus infections (Cheng *et al.*, 2021).

Heat shock proteins are well studied and abundantly expressed in insects in response to abiotic stressors such as temperature, ultraviolet radiation, drought and dehydration, anhydrobiosis, chemicals, metals, specific nutrients, injury adaptation, and hypoxia, as well as to the presence of virus‐derived double stranded RNA (Liu *et al.*, 2012; Zhao & Jones, 2012). Zhao & Jones (2012) comprehensively discussed the expression of *hsp* genes in mosquitoes, *D. melanogaster*, *A. mellifera*, and *Spodoptera* spp. as well as reviewed the stressors involved.

Small HSPs (Hsp22, Hsp23, Hsp26, and Hsp27) are well studied in *D. melanogaster* flies, where HSPs are induced by thermal changes (heat/cold), oxidative stress (anoxia), overcrowding, aging, as well as diapause (Fleming *et al.*, 1988; Holbrook *et al.*, 1995; King & MacRae, 2015). The proteins Hsp22, Hsp26, and Hsp27 were abundantly produced in aging *Drosophila* flies, evidently slowing down the aging process, and increasing lifespan by 30% (King & Tower, 1999; Wang *et al.*, 2004). Similar findings were seen when overexpressing *hsp22* in *Drosophila*, which increased life span by more than 30% and prevented early aging events by prolonging the pre‐mortality phase and increasing resistance to oxidative injuries and thermal stress (Morrow *et al.*, 2004). The *hsp27* gene was expressed in all tested tissues and developmental stages of the Asian honeybee *Apis cerana* (Fabricius) (Hymenoptera: Apidae), *w*hen exposed to heat shock, diverse chemicals such as H_2_O_2_, SO_2_, formaldehyde, ethanol, and pesticides, and when infected by the bacteria *Staphylococcus aureus* or *Micrococcus luteus* (Liu *et al.*, 2012).

Among the large heat shock proteins (Hsp60, Hsp70, and Hsp90), the *hsp70* gene showed an upregulation in the overwintering pupal diapause of the flesh fly *Sarcophaga crassipalpis* (Macquart) (Diptera: Sarcophagidae), implying the role of Hsp's in the survival ability of overwintering insects (Rinehart *et al.*, 2007). In the same fly, in response to hypoxia, there was also an upregulation of *hsp70* gene expression, followed by the upregulation of the *hsp18*, *hsp23*, and *hsp25*, *hsp40* and *hsp60* genes, but *hsp27* was only upregulated during anoxia (King & Macrae, 2015). In *D. melanogaster* flies incubated for 6 h in 0.5% oxygen (hypoxia), almost 80 genes were upregulated starting with *hsp68*, followed by *hsp23*, *hsp22* and *hsp67bc*, while milder hypoxia levels resulted in an increased transcription level for only 20 genes (Liu *et al.*, 2006). Also, *hsp70* and *hsp90* were strongly upregulated in response to sunlight in the Antarctic midge, *Belgica antarctica* (Jacobs) (Diptera: Chironomidae) (Lopez‐Martinez *et al.*, 2008).

### The antioxidant stress responses

Overall, various factors, such as irradiation, aging, thermal, hypoxic and reproductive stress, have been associated with the generation of ROS, causing oxidative damage to cells of insects (Aruoma, 1998; Dröge, 2002; Dubovskiy *et al.*, 2008; Lopez‐Martinez *et al.*, 2014; Michalkova *et al.*, 2014; Zhang *et al.*, 2021). Resistance to oxidative damage is highly linked to antioxidants, which act as enzymatic or nonenzymatic detoxifiers by scavenging on the ROS to reduce lipid peroxidation and decrease damage to nucleic acids and proteins (Lopez‐Martinez *et al.*, 2008; Michalkova *et al.*, 2014; Teets *et al.*, 2019). In insects, an array of antioxidant enzymes, including SOD, catalase (CAT), glutathione S‐transferase (GST), glutathione reductase (GR), peroxidase (POD), and glutathione peroxidase (GPX), act in response to endogenously produced oxidants and environmental stressors (Felton & Summers, 1995). SOD, CAT, and GPX have been termed as first‐line defense antioxidants that are repetitively generated against the superoxide anion radical (Ighodaro & Akinloye, 2018). Glutathione is essential in maintaining the physiological balance between pro‐oxidants (e.g., dioxin, paraquat quinones, 8‐methoxypsorlen, arsenic, and mercury) and antioxidants (Zabłocka & Janusz, 2008). The Sf9 and Tn‐5B1‐4 insect cell lines derived from the caterpillars *Spodoptera frugiperda* (Smith & Abbot), and *Trichoplusia ni* (Hübner), (both Lepidoptera: Noctuidae), contain the antioxidant enzymes manganese superoxide dismutase (MnSOD) and copper‐zinc superoxide dismutase (CuZnSOD), for the reduction of the superoxide radical (O_2_•−) to hydrogen peroxide (H_2_O_2_). These cells also contain ascorbate peroxidase (APOX) activity to reduce the resulting H_2_O_2_ to H_2_O (Wang *et al.*, 2001a). Both cell lines also contain GR and dehydroascorbic acid reductase activities for regenerating the reduced forms of glutathione and ascorbic acid, respectively (Wang *et al.*, 2001a). Irradiation experiments with the Sf9 insect cells suggest that lepidopteran insect cells carry a stronger antioxidant protection against radiation‐induced macromolecular damage, growth inhibition and cell death than mammalian cells (Suman *et al.*, 2015). The invasive Western flower thrips *Frankliniella occidentalis* (Pergande) (Thysanoptera: Thripidae), exhibited an upregulation of antioxidant genes and an increased enzyme activity (SOD, GST and POD), when exposed to thermal stress (Yuan *et al.*, 2021). Also, in the heat stress challenged lace bug, *Corythucha ciliata* (Say) (Hemiptera: Tingidae) the activities and concentrations of SOD, CAT, GR, and glutathione, as well as malondialdehyde (MDA), a marker for lipid peroxidation, increased significantly (Ju *et al.*, 2014).

Antioxidants in insects can also be induced and upregulated by biotic stress (pathogenic infections brought about by viruses, parasites, bacteria and fungi) (Wang *et al.*, 2001b; Pan *et al.*, 2012; Karthi *et al.*, 2018). Overexpression of genes for the antioxidant enzymes SOD, CAT, GPX, GST, glutaredoxin (Grx), thioredoxin (Trx), and protein disulfide isomerase (PDI) was detected following the infection of mosquito cells with dengue virus (Cheng *et al.*, 2021). On the first day following inoculation of wax moth larvae, *G. mellonella* with the bacterium *Bacillus thuringiensis*, increased enzyme activities of SOD, GST, malondialdehyde, and elevated ratios of concentrations of oxidated and reduced thiols (RSSR/RSH) were exhibited. (Dubovskiy *et al.*, 2008). The antioxidant MnSOD was also shown to significantly reduce oxidative damage in Tn‐5B1‐4 cells infected with the baculovirus Autographa californica multiple nucleopolyhedrovirus (AcMNPV) (Wang *et al.*, 2004). Contrastingly, infection of Sf9 and Tn‐5B1‐4 insect cell lines with the same baculovirus decreased the enzymatic activity of CuZnSOD (Wang *et al.*, 2001b). Also, the exposure of *S. litura* to the fungus *Aspergillus flavus* resulted in modification of the levels of antioxidant enzymes 48 h after exposure (Karthi *et al.*, 2018).

Also, hypoxic and anoxic conditioning during irradiation of fruit flies (currently a routine application in SIT), leads to redox regulation (the balance between oxidants and antioxidants), which has been proven to protect the Caribbean fruit fly *Anastrepha suspensa* (Loew) (Diptera: Tephritidae) from damaging free radicals, enhancing male sexual performance (Lopez‐Martinez & Hahn, 2012). On the contrary, the related Mexican fruit flies, *Anastrepha ludens* (Loew), that were exposed to hypoxia postproduction exhibited the same performance in the measured quality parameters, similar to that of control flies that were not exposed to hypoxia (Arredondo *et al.*, 2016, 2018). Our recent study has similarly shown that hypoxia during irradiation of tsetse flies, does not affect viral densities of GmmIV and GmmNegV as no significant differences were evident between the viral densities in flies irradiated under hypoxia compared to those irradiated under normoxia (Mirieri *et al.*, 2023), suggesting that, in some circumstances, oxygen deprivation may not make a difference in the processes (e.g., irradiation and transportation) involved in SIT.

Additionally, *G. morsitans morsitans* experiencing oxidative stress during reproduction, overcame the effects through the production of antioxidants (Denlinger & Ma, 1974; Michalkova *et al.*, 2014) as the antioxidant levels increased, leading to inhibition of reproductive senescence, hence allowing continued productivity during their entire lifetime (Michalkova *et al.*, 2014). Experimental overexpression of genes that counter aging in the fruit fly *D. melanogaster* resulted in the long‐lived flies, named Methuselah (Lin *et al.*, 1998; Rose *et al.*, 2004). Extension of lifespan in the Methuselah flies containing the overexpressed *mth* gene, enhanced the resistance to other forms of stress such as starvation, elevated temperature, dietary paraquat, and free radical generators; superoxide dismutase (SOD) and catalase (Lin *et al.*, 1998)

While antioxidants are protective against pathogenic infection in general, for other insects, such as tsetse flies, the addition of a range of antioxidants (glutathione, cysteine, N‐acetyl‐cysteine, ascorbic acid, and uric acid) in infectious blood meal inhibited the cell death of *Trypanosoma brucei brucei* ingested with the blood meal, parasites that would otherwise be cleared in the midgut (MacLeod *et al.*, 2007b). The addition of antioxidants (ascorbic acid and D‐N‐nitro‐arginine‐methyl‐ester) promoted the maturation of trypanosomes in the midgut but decreased the proportions of these infections maturating in the salivary glands (MacLeod *et al.*, 2007a, 2007b). Enhanced susceptibility of tsetse fly adults to trypanosomes will negatively affect SIT programs, because the released flies may succumb to trypanosome infections after feeding, or if they survive, participate in the transmission of trypanosomiasis to humans and animals.

## The immune response

### Introduction to immune responses against biotic stress in insects

Immune responses in insects are mainly triggered by biotic stressors, although they can also be triggered by abiotic stressors. In mass rearing of dipteran insects, biotic stress typically arises from viral, bacterial, parasitic, and fungal infections, some of which are introduced from the field during the beginning of colonies and some of which are inherently present and that possibly are triggered to overt infections by abiotic stressors such as humidity, temperature and crowding. In a number of insects, biotic stress originating from such infection is controlled by several layers of defense, involving physical barriers and immune responses (Rosales, 2017; Belachew, 2018). Immune responses are mediated by pathogen recognition receptors that recognize various stimuli (e.g., infections), resulting in the activation of various signaling pathways trigger specific immune responses, including cellular, peptide, and RNAi‐based defense mechanisms (Rosales, 2017). While there is scarcity of publications on immune responses toward infections in dipteran insects for mass rearing, there is sufficient information that can be postulated from the same insects (e.g., mosquitoes) in other environments. These are discussed in the following subsections with few examples from mass‐reared insects, with an aim to inform on molecular markers that could be used to carry out diagnostics and research in mass reared dipteran insects.

### Physical barriers

The major physical barriers in insects are the cuticle or the exoskeleton, the tracheal respiratory system, and the peritrophic membrane (PM) of the midgut (Merzendorfer & Zimoch, 2004; Lemaitre & Hoffmann, 2007; Baia‐da‐Silva *et al.*, 2018). When the physical barriers fail, the microbiological barriers set in to challenge infections. Microbiological barriers include the midgut microbiome in tsetse flies, which includes the bacteria *Wigglesworthia*, *Wolbachia*, and *Sodalis*. Those bacteria have mutualistic relationships with the tsetse flies and might confer immune protection to their hosts through antibacterial and antiviral effects and by affecting the redox homeostasis (Pan *et al.*, 2012; Wong *et al.*, 2015). *Wolbachia‐*infected *Ae. aegypti* mosquitoes showed increased levels of expression of genes related to immunity to counteract oxidative stress, where *Wolbachia*‐induced upregulation of ROS activated the Toll pathway resulting in antioxidant production. In turn the Toll pathway induced antimicrobial peptides, leading to reduced dengue virus susceptibility of *Wolbachia*‐infected mosquitoes (Pan *et al.*, 2012).

*Wigglesworthia* has been particularly implicated in promoting immunity against trypanosome infection in tsetse flies (resulting in trypanosome refractory flies) (Weiss *et al.*, 2013), and *Enterobacter* against *Plasmodium falciparum* infection in mosquitoes (Dennison *et al.*, 2016). The absence of the obligate symbiotic bacteria *Wigglesworthia* in colonized *Glossina* spp. has been found to compromise the immunity of tsetse fly larvae during intrauterine development (Weiss *et al.*, 2011). Tsetse flies that were immune‐stimulated with *Escherichia coli* bacteria, closely related to the natural *Sodalis* and *Wigglesworthia* tsetse fly gut endosymbionts, were able to effectively block trypanosome transmission, implying the role of endosymbionts in the immune response and protection (Hao *et al.*, 2001). *Wolbachia*’s immune protection in mosquitoes is evident for several pathogens including dengue virus, *Plasmodium*, and filarial nematodes (Pan *et al.*, 2012) and this endosymbiont is also mediating antiviral immunity in *Drosophila* (Palmer *et al.*, 2018). Several more studies show endosymbiont bacteria as acting in defense against viral infections, a vast number of these studies being on the role of *Wolbachia* in *Drosophila* and mosquitoes among other insects (Teixeira *et al.*, 2008; Pan *et al.*, 2012; Wong *et al.*, 2015; Pimentel *et al.*, 2021). The insect gut microbiome is also known to enhance the fitness of irradiated tephritid flies through improved copulation and increased mating competitiveness, among other quality indicators (Deutscher *et al.*, 2019).

### Pathogen recognition receptors

When the physical and microbiological barriers fail to prevent or control infections, then immune effector mechanisms may kick in (Jiang *et al.*, 2021). The insect immune response is a complex system, which involves the interaction of various cell types, molecules, and pathways that mediate immune responses to fight off infections (Rosales, 2017; Jiang, 2021). Immune responses may be broadly categorized as immune signaling pathways, cellular immune responses, and antiviral, antibacterial, and antifungal mechanisms mediated by antimicrobial peptides (AMPs) (Lemaitre & Hoffmann, 2007; Strand, 2008; Rosales, 2017). The antiviral, antibacterial, and antifungal defense mechanisms are also largely mediated by the immune signaling pathways hereafter discussed. Pathogen invasion is usually recognized by pathogen recognition receptors (PRR) such as the peptidoglycan recognition proteins (PGRPs), Gram‐negative binding proteins (GNBPs), and *β*‐glucan recognition proteins (*β*GRPs) for bacterial and the serine protease Persephone (Psh) for fungal infections. The binding of ligands to the PRRs may trigger various signaling pathways (see section *Pathogen recognition receptors*), which then leads to the production of effector molecules such as the AMPs expressed from the fat body (Rosales, 2017) (see section *Signaling pathways involved in immune responses*). In viral infection, dsRNA molecules are an important trigger for immune responses and stimulate RNA interference mechanisms (see section *Cellular immune responses*).

### Signaling pathways involved in immune responses

After detection of pathogens (especially bacteria and fungi) by PRRs, a series of immune signaling pathways are activated in insects to stimulate the required immune responses. These signaling pathways include the Toll and IMD pathways that both act through nuclear factor *κ*B (NF‐*κ*B, called Relish in *Drosophila*). Another pathway that is often triggered is characterized by the combined action of JAK and STAT. All these pathways are well described in *Drosophila* (Lemaitre & Hoffmann, 2007; Hetru & Hoffmann, 2009) and other insects (Cheng *et al.*, 2016). For a comparison of insect and mammalian signaling pathways, see Sheehan *et al.* (2018).

The Toll pathway (similar to the mammalian pathways depending on Toll‐like receptors (TLRs) is induced by viral, Gram‐positive bacterial, and fungal infections, while the IMD pathway (like the mammalian tumor necrosis factor, TNF) defends against Gram‐negative bacterial infections, as well as viral infections, as was observed in mosquitoes (Cheng *et al.*, 2016, 2021). The IMD pathway has also been implicated in defense against pathogenic trypanosome infections in tsetse flies (Hao *et al.*, 2001). The IMD and Toll pathways activate host gene transcription mediated by the transcription factors Relish, or the Dorsal and Dorsal‐related immune factors (Dives), respectively. These transcription factors activate genes needed for the production of AMPs. The Toll and IMD pathways also play a role in antiviral immunity with varying activity of the products of the activated genes against different insect‐specific viruses and arboviruses (Kingsolver *et al.*, 2013).

The JAK‐STAT pathway was first discovered as significant to human immunity, where the JAK proteins phosphorylate each other and later phosphorylate the STAT subunits, allowing them to move to the nucleus and bind to the promoters of their target genes (O'Shea & Plenge, 2012; Rosales, 2017). The JAK‐STAT pathway is known to be activated in response to insect‐specific pathogenic viruses and arboviruses vectored by mosquitoes. This pathway was first discovered in *Drosophila* infected with Drosophila C Virus (DCV) (Kingsolver *et al.*, 2013). In *Drosophila*, the JAK‐STAT pathway ligands consist of three cytokine‐like proteins known as unpaired (upd), upd2, and upd3 (Wright *et al.*, 2011), which bind to the single receptor Domeless (Dome), which in turn binds to a particular JAK (Hopscotch) which leads to phosphorylation of STAT92E, and as a consequence induces the production of cytokines and other immune response proteins (Rosales, 2017).

Evidence also shows that the Jun N‐terminal kinase (JNK) signaling pathway is significant in the immunity of insects. It has been determined as a genetic determinant of aging in *D. melanogaster*, functioning at the center of a signal transduction network that coordinates the induction of protective genes during an oxidative challenge, hence extending their lifespan (Wang *et al.*, 2003; Pan *et al.*, 2012)

Immune signaling pathways may act simultaneously, as one virus species may trigger several pathways in a single host, or several antiviral pathways may be induced at the same time, in one insect, by several viruses (Lamiable & Imler, 2014). In the silkworm, *Bombyx mori* (Linnaeus) (Lepidopters: Bombycidae), the roles of the NF‐*κ*B‐mediated, IMD, stimulator of interferon gene (STING) and the JAK/STAT pathways have been elucidated as antiviral mechanisms (Jiang, 2021). In mosquitoes, antiviral mechanisms involving phenol oxidase (PO), Toll, IMD, and the JAK‐STAT pathways, cellular and humoral responses, in addition to the extracellular pattern recognition mechanisms and thioester containing proteins (TEPs), are well documented (Fragkoudis *et al.*, 2009; Cheng *et al.*, 2016). The siRNA, Toll, and JAK‐STAT pathways were observed to function together with the gut microbial flora in limiting arboviral infection in the midgut of the mosquito (Cheng *et al.*, 2016). The RNAi and JAK‐STAT immune response pathways are both active in the midgut of *Ae. aegypti*, suggesting roles in controlling arboviral infection via blood meals, while the Toll pathway was shown to exert antiviral activity in a midgut‐specific manner in *Drosophila* and mosquitoes (Cheng *et al.*, 2016).

### Cellular immune responses

Insects have immune responses that rely on special cells called hemocytes. The main types of hemocytes are granulocytes, oenocytoides, and prohemocytes. Granulocytes help with engulfing harmful substances, oenocytoides assist with a process called melanization, and prohemocytes are important because they can develop into other blood cells (Strand, 2008; Pal & Kumar, 2014). Hemocytes are also involved in the production of AMPs, nodule formation, agglutination, and encapsulation of pathogens (Eleftherianos *et al.*, 2021). Large foreign objects such as parasitic wasp eggs are being encased with melanotic capsules composed of hemocytes in *Drosophila*, which in addition produce cytotoxic molecules as part of the cellular response (Nappi *et al.*, 2009). The immune challenge rapidly triggers phagocytosis and encapsulation (Bruno *et al.*, 2021; Eleftherianos *et al.*, 2021). Other cellular processes include apoptosis (programmed‐cell death) and autophagy (natural degradation of cellular components). These processes help to stop viruses from reproducing and spreading in infected insects (Lamiable & Imler, 2014). Extracellular complement‐like proteins that limit flavivirus infection in *Ae. aegypti* by inducing the production of AMPs by hemocytes have also been reported (Xiao *et al.*, 2014). Melanization is also an important protecting, cellular immune response that produces melanin and deposits this pigment on invading pathogens (Christensen *et al.*, 2005).

### Peptide‐based defense mechanisms

Insects are a rich source of AMPs for their own defense, but also for potential pharmaceutical use (Albiol‐Matanic & Castilla, 2004; Yi *et al.*, 2014). These AMPs are mainly made in the fat body and are divided into four groups based on their chemical structure and specific features: the α‐helical (cecropin and moricin), the cysteine‐rich (insect defensins and drosomycin), the proline‐rich (apidaecin, drosocin, and lebocin), and the glycine‐rich (attacin and gloverin) peptides. Among these AMPs, cecropins, defensins, proline‐rich peptides, and attacins are the most common (Lemaitre & Hoffmann, 2007; Yi *et al.*, 2014; Rosales, 2017). AMPs are effector molecules causing a range of antibacterial, antifungal, and antiparasitic activities, including membrane disruption, interference with bacterial metabolisms and targeting of cytoplasmic components (Wu *et al.*, 2018). More recently, antiviral peptides (AVPs) have been found that show a great potential in defending against viral attacks, as observed in *D. melanogaster* and *B. mori* (Yi *et al.*, 2014; Feng *et al.*, 2020). Insect AVPs include cecropin, melittin, alloferon 1 and 2, myristoylated‐peptides, TNGiv 1 and 2, attacin C, diptericin C, and C‐lysozyme (Feng *et al.*, 2020).

The induction of several host genes for AMP synthesis overlaps between infections with viruses, bacteria, and fungi (see Table 1). AMPs (and hemocytes) help prepare the immune system for future infections (immune priming). After insects are exposed to pathogens for the first time, they produce more of these antimicrobial peptides (Sheehan *et al.*, 2020). Black soldier flies, *Hermetia illucens* (Linnaeus) (Diptera: Stratiomyidae), feed on decaying organic materials and can be exposed to many germs. To protect themselves against infections they process many AMPs, 57 of which were identified in a single study (Moretta *et al.*, 2020). In tsetse flies, the identified AMPs (attacin, defensin, and diptericin) produced in fat body tissue are dependent on the type of pathogen involved: The scala of AMPs induced by *E. coli* infections differ from those found after trypanosome exposure. Moreover, the molecular signals leading to AMP synthesis differ for the various life stages of trypanosomes, resulting in deviations in AMP expression patterns (Hao *et al.*, 2001). These variations are functionally important, as the expression of attacin reduced trypanosome infections, but did not affect the Gram‐negative bacterium *Sodalis glossinidius* (Hu & Aksoy, 2005). In his review, Harrington (2011) described in detail the role of trypanocidal AMPs in the immune response of the tsetse fly against trypanosomes, as well as the therapeutic actions of antimicrobial peptides against Human African trypanosomiasis (HAT).

### RNA interference (RNAi)

Insects use a key method to fight viruses called RNAi. This mechanism works by stopping or breaking down viral (m) RNA, using short RNA molecules that complementarily match the viral sequences (Jiang, 2021). The RNAi pathway is a vigorous antiviral mechanism, mediated mainly through small interfering (si)RNA generated in the cell from dsRNA replication intermediates, but also involving micro‐RNA (miRNAs) and piwi‐interacting RNA (piRNA) (Meki *et al.*, 2018; Gamez *et al.*, 2020a). The RNAi response is highly conserved in insects and plays an important role in antiviral strategies, as also observed in various dipterans, including *Drosophila*, tsetse flies, and mosquitoes (Zambon *et al.*, 2006; Cheng *et al.*, 2016; Meki *et al.*, 2018). RNAi is working against RNA and DNA viruses (Kingsolver *et al.*, 2013; Cheng *et al.*, 2016; Meki *et al.*, 2018). Hemocoel injections of tsetse flies with the DNA virus GpSGHV upregulated three key RNAi genes: AGO1, AGO2, and DCR2 implying the role of RNAi in controlling this DNA virus (Meki *et al.*, 2018). Since many extensive reviews are available on RNAi in insects, we do not further elaborate here.

## Interactions between stress and responses

Often different stressors act simultaneously on a single individual and then these stressors may very well alter the immune response that would normally be mounted in the presence of a single stressor. Stressors may directly influence the effect of other stressors or lead to trade‐offs, such as diet restrictions extending the lifespan at the cost of reproduction in *C. capitata* (Davies *et al.*, 2005). Nutritional stress in female tsetse flies resulted in pupae with lower weight, and the next generations had a lower fat content and showed a reduction in AMP gene expression levels. Consequently, the offspring of the starved flies were more susceptible to infections with trypanosomes (Akoda *et al.*, 2009). Also, as reported for the bumblebee (*B. terrestris*), ovary activity significantly negatively reduced their longevity, since resources were directed toward their reproductive capacity (Blacher *et al.*, 2017).

Immune responses are often mounted at energetic and physiological costs (e.g., the metabolic rate), as seen in a wide range of insects (Ardia *et al.*, 2012), and the same most likely applies to stress responses. Sometimes these responses act independently, such as in the lepidopteran maize pest *Ostrinia furnacalis* (Guenée) (Lepidoptera: Cambridae), where heat (40 °C) mainly triggered stress response genes, while cold conditions (8 °C) upregulated immune response genes (Chen *et al.*, 2019a). This phenomenon allows the insect to survive under highly variable and extreme temperature conditions. In *Ae. aegypti*, genes involved in immune responses against Chikungunya virus infection were not upregulated at stressful elevated temperatures (Wimalasiri‐Yapa *et al.*, 2021). Also in many biological systems, prolonged stressful circumstances lead to an increased susceptibility to pathogenic infection as consequence of reduced expression of immune‐related genes (Sachdev *et al.*, 2017; Lecocq *et al.*, 2019). Such susceptibility may directly result from effectors of stress (e.g., stress hormones) diverting molecular resources away from immune functions. For example, lipid transport proteins, which are involved in both stress and immune responses, can be diverted from an immune response to a stress response (e.g., in fight or flight behavior), leading to reduced resistance to pathogenic infections as shown in various insects (Adamo, 2017).

For a number of insects that experience dense conditions in their natural life, density‐dependent polyphenisms may be observed (e.g., darkening of the skin caused by phenoloxidase expression). These insects appear to have mechanisms in place that modulate hormonal and cellular responses during overcrowding to provide them with prophylactic immunity, as reported for instance, in the rice ear‐cutting caterpillar *Mythimna separata* (Walker) (Lepidoptera: Noctuidae) (Kong *et al.*, 2018). Such prophylactic immunity probably makes them less vulnerable for infections during mass‐rearing. In addition, high population densities in mass‐reared insects can cause vulnerability to other stressors and altered immune responses (Akoda *et al.*, 2009; Lalouette *et al.*, 2011; Krautz *et al.*, 2014; Sachdev *et al.*, 2017; Wojda, 2017). As insects age, their ability to resist stress declines. In aging fruit flies, this decline is counteracted by increasing the levels of the Hsp22 protein, which extended the fly's lifespan by improving mitochondrial functioning (Kim & Kim, 2010). On the other hand, stress is not always disadvantageous or with negative consequences to insects. For instance, while stress normally weakens the immune system of most organisms, Adamo (2017) found that stress may induce a pro‐inflammatory state that boosts early immune responses in insects. This author also found that, intense, short‐term exposure to stress hormones is necessary for the reconstruction of the immune system of insects during their “flight or fight” behavior (Adamo, 2014). Further on positive effects, mild stress caused by short hypergravity exposure, increased the longevity of *D. melanogaster* (Bourg *et al.*, 2000) while low temperatures induced specific expression of immune response genes in *O. furnacali*s (Chen *et al.*, 2019a). Mild irradiation and sometimes optimum irradiation doses have been found to increase the survival of mass reared dipteran flies. Tsetse fly pupae irradiated at 29 d of age survived the longest period compared to controls (Mirieri *et al.*, 2024) Irradiation with X‐rays of *D. melanogaster* at 1.2 and 2.1 Gy resulted in approximately 12% increase of male life span, and the mean fecundity of females did not differ from the control females at these doses (Vaiserman *et al.*, 2004). For purposes of SIT, an experiment on radiation dose fractionation in adult *Ae. aegypti* mosquitoes showed improved longevity of the flies (Yamada *et al.*, 2023). In addition, a brief exposure to hypoxia/anoxia (forms of stress following oxygen deprivation) before irradiation induced radio‐protective effects, on the survival of mosquitoes (Yamada *et al.*, 2022). Also, irradiation in a nitrogen atmosphere that displaced oxygen maintained and even enhanced the survival of tsetse flies (Langley *et al.*, 1974; Vreysen & de Vloedt, 1995). In fruit fly mass rearing, protocols (FAO/IAEA/USDA, 2019) require that the flies are subjected to oxygen depletion (anoxia/ hypoxia) to reduce the impact of irradiation on quality parameters, a decision following several optimization experiments (Nestel *et al.*, 2007). Males sterilized after 1 h of hypoxic conditions were significantly more competitive against fertile males, compared to flies irradiated under normoxic conditions, a significant finding for SIT (Tussey *et al.*, 2022).

## Conclusions and directions for future research

This review discussed abiotic and biotic stressors, the subsequent stress and immune responses, and the way these interact in insects, providing grounds for improving the rearing conditions for SIT insects. In summary, the host's immune response system mainly acts as a response to biotic stress from infection by pathogens, which activate various pathways that regulate genes of the immune system (see Table 1 on the examples of stressors, induced genes, and important signaling pathways involved in insects). Abiotic stress can influence immune functions by enhancing or suppressing immunity, depending on the context, the stressor, the duration of the stress, and the concentration of stress hormones (Ottaviani & Franceschi, 1996; Adamo, 2008; Nunes *et al.*, 2021). Stress responses to biotic and abiotic factors, may overlap with immune response systems, because often, the same genes and pathways respond to both stress (abiotic) and (biotic) infection (Adamo, 2008, 2010). Stress factors such as temperature, humidity, and nutrition quality and deprivation may be the cause for the reactivation of asymptomatic (latent) infections in mass‐rearing systems (Abd‐Alla *et al.*, 2010; Jiang *et al.*, 2021). Temperature is a well investigated stressor in insects, especially in mosquitoes that transmit arboviruses, as temperature is fundamental for survival of insects and the severity of (arbo‐)virus infections (Bellone & Failloux, 2020; Wimalasiri‐Yapa *et al.*, 2021). A pathogenic infection normally induces an immune response in the host (as a biotic stressor) and the pathogen is often involved in an arms race with the host's immune system, as the host strives to eliminate the pathogen and the pathogen attempts to silence the host's immune system to increase its chances of survival in the host (Kariithi *et al.*, 2018). As a result, an induced immune response may speed up metabolism and lower antimicrobial activity. To fully understand the synergy of decoupling effect of immune insults like wounding, it is important to study a wide range of immune responses in a variety of insect species. Exposure to a low level of stress appears to be important in priming immunity and stress responses in insects (Ottaviani & Franceschi, 1996; Ardia *et al.*, 2012; Zhao & Jones, 2012; Cooper & Eleftherianos, 2017), since stress‐related biomolecules may “induce re‐configuration of networks at molecular, cellular, and physiological levels, allowing the organism to maintain optimal capacity to respond adequately to an internal changing environment” as phrased by Adamo (2012).

Notably, a vast majority of immune response studies can be found in dipteran systems, especially within *D. melanogaster* and mosquitoes, given their significance as model and medically important insects, respectively. However, a similar intensity of studies is yet to take place regarding tsetse flies, especially concerning antiviral immunity. Further, despite the wealth and diversity of the research summarized in this review of literature, we still have a limited view on the impact of all these stress factors and their responses (immune and stress‐related) in mass‐rearing systems for SIT programs and their implications for effective management of tsetse flies, fruit flies, and mosquitoes. For instance, there are limited investigations on the stress response mechanisms (genes and pathways) that lead to the activation of latent pathogen infections. There is also a lack of tools for diagnosing and establishing stress tolerance levels in dipteran insects under mass‐production for SIT. Empirical tests of the relationships between immunological reactions and the level of resistance to a range of stressors and pathogens may identify genes, whose expression can act as evidence of stress and antistress mechanisms leading to predictions of population dynamics in mass‐rearing facilities. Such tests can demonstrate, whether for instance, *Wolbachia* can be integrated into SIT programs as a protective agent against biotic stressors, as hinted at during an investigation on viral infections in insects carrying this symbiont (Zabalou *et al.*, 2004; Pimentel *et al.*, 2021). The identified genes that are correlated with stress tolerance levels can be tested as potential therapeutic targets for the control of stress levels in dipteran SIT insects (Wang *et al.*, 2001b; Giannakou *et al.*, 2004; Morrow *et al.*, 2004; Teets *et al.*, 2019). For example, since overcrowding is not a problem for social insects such as bees, wasps, and ants, and insects in general that show density‐related prophylactic immunity, researchers could use the knowledge on the genes responsible for this behavior in social insects to upregulate the corresponding genes as a prophylactic measure to induce disease resistance in SIT insect mass‐production systems (Barnes & Siva‐Jothy, 2000; Wilson *et al.*, 2002). Lessons can also be drawn from studies on social immunity for colonized insects to prevent activation and transmission of insect viruses (Cremer *et al.*, 2007). Antimicrobial peptides, genes, pathways, and microbiological barriers may be explored to find targets for the treatment of infections in colonies similar to pharmaceutical companies (Netea *et al.*, 2004; Teixeira *et al.*, 2008; Yi *et al.*, 2014; Meki *et al.*, 2018). Such identified genes may be explored in improving the productivity and survival of flies under mass‐production, given that SIT requires large numbers of flies for release. Finally, since multiples stress factors may occur simultaneously in an insect and hence the expression of the response genes can overlaps, routine screening for multiple factors, will be important to get a clear picture of the underlying, stress‐related problems during mass‐production and postproduction processes that together impact the fitness and performance of SIT insects. In view of the aforementioned knowledge gaps and the suggestions on filling them future research may endeavor to fill these gaps for a better understanding of stress in dipteran mass‐reared insects. We have therefore summarized immune and stress response pathways and genes that may be used in future research toward better management of insects under mass‐production and for the successful implementation of SIT programs (Table 1).

## Disclosure

The authors declare no conflict of interest.
